# Tryptophan metabolism and gut flora profile in different soybean protein induced enteritis of pearl gentian groupers

**DOI:** 10.3389/fnut.2022.1014502

**Published:** 2022-12-19

**Authors:** Wei Zhang, Aobo Pang, Beiping Tan, Yu Xin, Yu Liu, Ruitao Xie, Haitao Zhang, Qihui Yang, Junming Deng, Shuyan Chi

**Affiliations:** ^1^Laboratory of Aquatic Animal Nutrition and Feed, College of Fisheries, Guangdong Ocean University, Zhanjiang, Guangdong, China; ^2^Aquatic Animals Precision Nutrition and High Efficiency Feed Engineering Research Center of Guangdong Province, Zhanjiang, Guangdong, China; ^3^Key Laboratory of Aquatic, Livestock and Poultry Feed Science and Technology in South China, Ministry of Agriculture, Zhanjiang, Guangdong, China

**Keywords:** *Epinephelus fuscoguttatus♀ × E. lanceolatus♂*, soy meal, gut flora, tryptophan metabolism, enteritis

## Abstract

The substitution of high-level soy meals for fish meal (FM) generally leads to fish enteritis, accompanied by significant variations in gut flora. Relevant studies have pointed out a close relationship between tryptophan metabolism mediated by gut flora and vertebrate inflammatory bowel disease. Present study examines the role of tryptophan metabolism and gut flora profile in fish enteritis caused by different soybean meals. The 960 groupers were randomly assigned into 4 groups (*n* = 4), which including: (1) FM (the control group, fed with 50% FM feed), (2) SBM40 (replacing 40% FM with soybean meal), (3) SPC40 (replacing 40% FM with soybean protein concentrate), and (4) FSBM40 (replacing 40% FM with fermented soybean meal). Under average temperature and natural light, the groupers were cultivated with feeds of iso-nitrogen and iso-lipid for 10 weeks. The results showed that soybean meal feeds at all experimental levels had negative effects on fish gut physiology and growth performance. Typical enteritis features and fluctuations of immune system occur, which can be observed in the enzyme activities of total superoxide dismutase and lysozyme and in the contents of immunoglobulin M, complement 3 and complement 4. 16SrDNA high-throughput sequencing indicated that it greatly influenced the gut flora with the abundance of maleficent bacteria, like Vibrio, amplified with increasing dietary soybean meals. According to the “3 + 2” full-length transcriptome sequencing, soy meals at the three experimental levels inhibited the key gene expressions of tryptophan metabolic pathway in fish gut, however, there are some differences in the types of key genes that are inhibited. The canonical correlation analysis showed that the changes in key gene expressions in tryptophan metabolic pathway had a positive correlation with the expressions of pro-inflammatory genes (*P* < 0.05) and negatively correlated with the expression of anti-inflammatory genes (*P* < 0.05). It is speculated from this study that tryptophan metabolism is closely related to fish soy meal-related enteritis, and the abnormal tryptophan metabolism caused by intestinal flora imbalance may play an important role. In the future research, we can further study the tolerance of fish to soy meals feed from two aspects of tryptophan metabolism and intestinal flora changes.

## Introduction

In recent years, the total amount of aquaculture has continued to increase, but the fish meal (FM) output almost remained unchanged. Since FM presents to be a key source of protein for aquaculture, aquaculture farmers urgently need new and sustainable alternatives for it (1). Thus, attention has been paid to plant materials during the procedure. Among them, soy products are considered one of the most promising FM substitutes because of their high yield and relatively balanced amino acid composition (2). However, soy products with high substitution levels, especially soybean meal (SBM), are easy to cause gut mucosal damage and enteritis in fish, which seriously endangers the gut health of fish, and limits its extensive application in aquaculture industry.

According to former research, the influence of SBM on fish enteritis varies with its anti-nutritional factors (ANFs), including phytates, protease inhibitors, saponins, lectins, phytosterols, and oligosaccharides (3). The histological characteristics of SBM induced enteritis (SBMIE) in fish include infiltration of different inflammatory cells, the swelling submucosa and lamina propria, the shortening of mucosal fold, and a decrease in absorption vacuoles of gut epithelial cell (4). It has been found that high-level SBM has negative impact on the gut health of a variety of fish, such as zebrafish, carp, rainbow trout, and Atlantic salmon (5–7). Fish SBMIE mainly occurs in the hindgut, a crucial part of protein absorption by endocytosis and is more susceptible to food stimulation, causing intestinal diseases (8). In order to reduce the antigen content, the SBM is often processed into different product types.

In aquatic feed, soy products used mainly include SBM, soybean protein concentrate (SPC), as well as fermented soybean meal (FSBM). SBM, a by-product of soybean oil extraction, contained various ANFs mentioned above. SPC is a product made from soy by extracting oil and low molecular weight soluble protein components by alcohol. Compared with SBM, SPC removed most ANFs and is a good plant protein source in the fish and shrimp diet (9). However, the solubility of SPC extracted by the alcohol method is decreased, and the nitrogen solubility index is reduced to about 10%, which limits its application (10). FSBM refers to a protein material prepared by fermentation of SBM by microorganisms. Fermentation of microorganism significantly purifies the content of ANFs in SBM, and decomposes soybean protein into small molecular proteins, small peptides, and amino acids, thus improving the digestion and absorption abilities (11). However, at present, due to the different fermentation technology, there are also many problems in FSBM, such as the lack of strict standards and specifications, and its product quality is still uneven (12).

Previous studies on fish SBMIE mostly focused on the influence of ANFs in SBM on gut health. But researchers found that the role of ANFs is complex, such as fish enteritis induced by soy saponins may be related to the basal protein source (13); or dose-dependent and independent of the basal protein source (14); or it is a secondary effect on the interference characteristics of cell membrane, and the SBMIE is mainly induced by external antigens (15). According to existing research, no clear results about the pathogenic mechanism of fish SBMIE have been made, but researchers generally believe that the gut flora play an important role during this process (16–18).

Tryptophan is an essential amino acid. In recent years, lysine and methionine have been widely used in formula diets, making tryptophan the main limiting amino acid in diet (19). In aquaculture, researchers found that tryptophan can promote the growth and gut development of carp and shrimp, regulate the gut microecological balance, and improve the survival rate (SR) of groupers (20–22). The related studies have shown the benefits of tryptophan on the gut health of aquaculture objects, but there are few reports on the fish SBMIE. However, in mammals, the relationship between tryptophan metabolism and human inflammatory bowel disease has become a research hotspot in recent years (23, 24). In present study, we also found that different soy meal substitution for FM at high levels, resulting in abnormal tryptophan metabolism in the gut tract of pearl gentian grouper.

Pearl gentian grouper is a typical marine carnivorous fish and is the important cultivated varieties in China and many places in the world. Its meat is tender, grows fast, has strong disease resistance, obvious hybridization advantages, and good market price. It can also be used as an ornamental fish, and has a broad market prospect. This study analysed the effects of different soy meals with high substitution levels on the growth physiology and gut tryptophan metabolism pathway of pearl gentian grouper, and preliminary analysed the gut flora profile variations. Present research offers a theoretical basis for the study of gut health problems caused by the substitution for FM with high level of plant proteins.

## Materials and methods

### Experimental diets

The experimental diets and their chemical compositions are shown in Table 1. The red FM, produced by Corporación Pesquera Inca S.A.C. at Bayovar Plant in Peru, contains 72.53% crude protein. The SBM (48.92% crude protein) and SPC (70.72% crude protein) were purchased from Zhanjiang Haibao Feed (Zhanjiang, China). The FSBM, containing 60.75% crude protein, was purchased from Xijie Foshan Co. Ltd. (Foshan, China). Four experimental diets of iso-lipid (about 10% total lipid) and iso-nitrogen (about 50% crude protein) were used by SBM, SPC, and FSBM to substitute 40% of FM protein; these diets were designated as FM (the control group), SBM40, SPC40, and FSBM40, respectively. To achieve amino acid balance, the experimental feeds were mixed with methionine and lysine (25). The specific preparation and storage methods of the four kinds of feeds were explained as below: first, the researchers ground the raw materials into powder, then screened it with 60-mesh sieves and weighed the following formula, before using the sequential expansion method to mix the micro constituents evenly. Second, the researchers added lipids and deionised water into it and then stirred it to get a fine mixture. Afterward, the feeds were air-dried to 10% humidity using pelletisers with diameters of 2.0 and 3.0 mm, respectively. The experimental diets were then conserved in sealed plastic bags at −20°C (26).

**TABLE 1:** Formulation and proximate composition of the experimental diets (%, dry matter).

Ingredients (%)	Diets			
	FM	SBM40	SPC40	FSBM40
Red fish meal	50.00	30.00	30.00	30.00
Soybean meal	0.00	29.65	21.74	23.89
Vital wheat gluten	5.00	5.00	5.00	5.00
Wheat flour	18.00	18.00	18.00	18.00
Casein	4.60	4.60	4.60	4.60
Gelatin	1.00	1.00	1.00	1.00
Fish oil	3.02	4.48	4.41	4.49
Soybean oil	2.00	2.00	2.00	2.00
Soybean lecithin	2.00	2.00	2.00	2.00
Microcrystalline cellulose	11.48	0.00	8.14	5.84
Calcium monophosphate	1.50	1.50	1.50	1.50
Ascorbic acid	0.05	0.05	0.05	0.05
Choline chloride	0.50	0.50	0.50	0.50
Vitamin premix^a^	0.30	0.30	0.30	0.30
Mineral premix^b^	0.50	0.50	0.50	0.50
Ethoxyquin	0.05	0.05	0.05	0.05
Lysine^c^	0.00	0.24	0.05	0.13
Methionine^c^	0.00	0.13	0.16	0.15
**Proximate composition (%, dry matter)**				
Crude protein	50.97	50.85	50.63	50.45
Crude lipid	10.15	10.44	10.71	10.54

^a^Vitamin premix consisted of (g/kg premix): VB_1_ 17.00 g, VB_2_ 16.67 g, VB_6_ 33.33 g, VB_12_ 0.07 g, VK 3.33 g, VE 66.00 g, retinyl acetate 6.67 g, VD 33.33 g, nicotinic acid 67.33 g, D-calcium pantothenate 40.67 g, biotin 16.67 g, folic acid 4.17 g, inositol 102.04 g, and cellulose 592.72 g.

^b^Mineral premix consisted of (g/kg premix): FeSO_4_⋅H_2_O 18.785 g, ZnSO_4_⋅H_2_O 32.0991 g, MgSO_4_⋅H_2_O 65.1927 g, CuSO_5_⋅5H_2_O 11.0721 g, CoCl_2_⋅6H_2_O (10%) 5.5555 g, KIO_3_ 0.0213 g, KCl 22.7411 g, Na_2_SeO_3_ (10%) 0.5555 g, and zeolite powder 843.9777 g.

^c^Lysine and methionine were added to balance amino acid with FM control group.

### Feeding experiment design

The healthy juvenile groupers, which weighed about 9 g, were bought from a commercial hatchery in Zhanjiang City, Guangdong, China. Before the experiment, 960 groupers were given 1 week to adapt to the experimental conditions, after which they were offered nothing to eat for a day. The researchers then anaesthetised the groupers with eugenol and divided them into four groups. According to size, fishes were randomly placed in 1,000 L cylindrical glass tanks. Every tank contained 60 fish. The experimental feeds were offered from 8:00 a.m. to 4:00 p.m. every day for 10 weeks, with four replicates. The uneaten feeds were siphoned to calculate the weight of the intake feeds, and the feed consumption ratio was determined (27). The feeding experiment was conducted within inner cultural systems belonging to the Marine Biological Research Base of Zhanjiang City (Guangdong, China). To ensure saturated oxygen concentration, air stones were used to continuously inflate air into containers. The experimental environment in containers involved a natural light cycle and a temperature of 29 ± 1°C. The concentrations of ammonia and nitrate were both lower than 0.03 mg L^–1^, and the dissolved oxygen involved in this study was ≥7 mg L^–1^. Within the first 14 days, 60% of the water in each container was exchanged every day, and after that, nearly 100% of the water was exchanged every day.

### Sample collection

The study was supported by the Expert Committee of the Fisheries College of Guangdong Ocean University. The research methods followed all applicable regulations and guidelines. Before sampling, the studied groupers were starved for 24 h. Then, they were anaesthetised with eugenol (1:10,000). After counting and weighing all fish in each tank, their specific growth rate (SGR), weight gain rate (WGR), hepatosomatic index (HSI), SR, and feed conversion ratio (FCR) were determined. To determine the enzyme activity, the researchers randomly selected six groupers from each water tank, drew blood from their tail veins, and stored it at 4°C for a night, before using a centrifuge at 3,500 rpm for 10 min to obtain serum. The blood was then stored at −80°C, preparing for analysing the enzyme activity. The hindgut tissues were excised, and any mesenteric and adipose tissues were removed. Hindgut samples were cut into small pieces and put in test tubes filled with RNAlater at 4°C for a night before being stored at −80°C for gene expression analysis.

For gut flora and transcriptome sequencing, the researchers randomly chose eight groupers. The hindgut tissue samples were obtained as mentioned above and placed in cryopreservation tubes, then directly put into liquid nitrogen, half of which was employed for 16S high-throughput sequencing, while the rest for transcriptome sequencing.


$$\mathrm{Weight}\mathrm{gain}\mathrm{rate}\left(\mathrm{WGR},\%\right)=\frac{100\times \left(\mathrm{final}\,\mathrm{body}\,\mathrm{weight}-\mathrm{initial}\,\mathrm{body}\,\mathrm{weight}\right)}{\mathrm{initial}\,\mathrm{body}\,\mathrm{weight}}$$

$$\mathrm{Specific}\mathrm{growth}\mathrm{rate}\left(\mathrm{SGR},\%/d\right)=\frac{100\times \left[\mathrm{Ln}\,\left(\mathrm{final}\,\mathrm{body}\,\mathrm{weight}\right)-\mathrm{Ln}\,\left(\mathrm{initial}\,\mathrm{body}\,\mathrm{weight}\right)\right]}{\text{days}}$$

$$\mathrm{Feed}\,\mathrm{conversion}\,\mathrm{ratio}\,\left(\mathrm{FCR}\right)=\frac{\mathrm{feed}\,\mathrm{intake}}{(\mathrm{final}\mathrm{body}\mathrm{weight}\mathrm{io}\left(\mathrm{FCR}\right)n(\mathrm{initi}}$$

$$\mathrm{Hepatosomatic}\mathrm{index}\left(\mathrm{HSI},\%\right)=100\times \left(\frac{\mathrm{hepatic}\,\mathrm{weight}}{\mathrm{body}\,\mathrm{weight}}\right)$$

$$\mathrm{Survival}\mathrm{rate}\left(\mathrm{SR},\%\right)=100\times \left(\frac{\mathrm{final}\,\mathrm{fish}\,\mathrm{number}}{\mathrm{initial}\,\mathrm{fish}\,\mathrm{number}}\right).$$

### Histological observation of enteritis

After the diet experiment, three fish were randomly chosen from every container. The DI sample was stored in 4% paraformaldehyde universal tissue fixative, which belongs to the Servicebio Technology Company of Wuhan, from China, for 24 h before being H&E stained. The stained sections were observed and photographed under 10 × 10 times microscopically (Olympus CKX41 microscope, Tokyo, Japan).

### Analysis of physiological indexes

In the present study, the researchers employed the BCA method to analyse the gut and serum samples (Beyotime Biotechnology Co., Ltd., Shanghai, China).

The enzyme activities of lysozyme (LYS) in serum and total superoxide dismutase (T-SOD) in hindgut were measured by fish ELISA kits, which were then employed to determine immunoglobulin M (IgM) content, complement 3 (C3) content, and complement 4 (C4) content in hindgut tissues. The testing kits were products of Shanghai Jianglai Biotechnology Co., Ltd. (Shanghai, China). The methods were performed according to the operating instructions in detail.

### Immune-related gene expressions

TRIzol kits (Invitrogen, Carlsbad, CA, USA) were applied to test the total RNA of the hindgut tissues. An Evo M-MLV reverse transcription kit (Takara, Japan) was employed to prepare qualified RNA samples to cDNA. They were then stored at −20°C for use. The primers were designed by Shenggong Bioengineering Co., Ltd. (Shanghai, China). The researchers obtained the template sequences of these primers from the sequencing of the “3 + 2” full-length transcriptome of the hindgut tissues of the studied groupers within the lab. The original data were recorded in the NCBI Sequential Read Archive (SRA) with accession numbers PRJNA664623 and PRJNA66441. Primers carried out by Primer Premier 5.0 software for pro-inflammatory genes were interleukin (*IL*)*1β*, *IL12*, *IL17*, *IL32*, and tumour necrosis factor (*TNF*)α, and anti-inflammatory genes were *IL4*, *IL5*, *IL10*, and transforming growth factor (*TGF*)*β1* (Table 2). The β-actin is presented to be the internal reference gene. The gene expressions were examined with RT-qPCR (Mastercycler ep realplex, Eppendorf, Germany). The PCR reaction environment were 95°C for 2 min with 1 cycle, 95°C for 15 s, 60°C for 10 s and 72°C for 20 s with 40 cycles, respectively. The target gene expressions were calculated using the 2^–ΔΔ^ ^Ct^ method.

**TABLE 2:** PCR primers for gut immune-related genes of grouper.

Gene	Forward 5′-3′	Revise 3′-5′	Annealing temperature (°C)	Primer efficiency (%)	Size (bp)
*IL1β*	AAGGTGGACGCCAACAGACA	GTTCACTGCAGGCTCAGGGA	56	95	153
*IL17*	GAGAGGACGGTGTCTGTGTGG	CATGCACAGTTGAGGGTGTGG	58	98	101
*p65*	TCAACCCAGTCCAAGCAGCA	GATGCTGCCAGCTGAACGTC	63	96	107
*IκBα*	ATGCAAAGGAGCAGCGTAACG	GAGGTTGGGGTCTGCTCCT	62	99	107
*TNFα*	AACTGTGTGTCCCCACTGCC	CCACAGATGGCCCAGGTCAT	58	95	81
*IL5*	GGCCAACAGTCAAGATGTCTGCC	GAATGACCAGGAGCAGTTCAGTGT	59	98	160
*IL10*	ACACAGCGCTGCTAGACGAG	TAGACTTGTGCCACGACGGG	66	94	142
*TGFβ1*	CTTCTCCTCCTCCTCGCTGC	GATGTTGCTGAGGGCTTCGC	62	99	195
*β-actin*	GGCTACTCCTTCACCACCACA	TCTCCAAGGCAACGGGTCT	65	99	188

*IL*, interleukin; *TNF*, tumour necrosis factor; *p*65, NF-κB-*p*65; *IκBα*, I-kappa-B-alpha; *TGF*, transforming growth factor.

### 16S high-throughput analysis of intestinal flora

According to the operation instructions, E.Z.N.A.™ Kit (Omega Bio-Tek, Norcross, GA, USA) was applied to extract the total RNA from the DI flora. Gene Denovo Co., Ltd. (Guangzhou, China) assisted in sequence detection and data analysis. The original data were recorded in the NCBI SRA, with the accession number PRJNA666309. The Supplementary File included specified analysis procedures.

### Transcriptome sequencing and KEGG annotation

Through the PacBio Sequel and Illumina HiSeq™ 4000 platforms, the “3 + 2” full-length transcriptome sequencing was carried out. Gene Denovo Co., Ltd. (Guangzhou, China) completed the sequencing process. The original data of the sequencing are stored as mentioned above. The gene expressions of | log2FC| > 1 and *P* < 0.05 were classified as differential genes (DEGs). The researchers put the DEGs gene into the KEGG database and carried out an in-depth analysis of the significantly activated signal pathways, which were relevant to signal transduction, infectious diseases and immune diseases/systems (*P* < 0.05).

### Validation of the tryptophan metabolism-related genes by RT-qPCR

To validate the accuracy of the “3 + 2” full-length transcriptome data, 10 DEGs from the tryptophan metabolism pathway were chosen for RT-qPCR: *AO*, *DDC*, *IDO2*, *AOX1*, *ALDH*, *DAO1*, *KYN*, *KAT1*, *CYP1A1*, and *LAAO*. The primer templates were the same as those mentioned above, and the primers are presented in Table 3.

**TABLE 3:** PCR primers for gut tryptophan metabolism pathway-related genes of grouper.

Gene	Forward 5′-3′	Revise 3′-5′	Annealing temperature (°C)	Primer efficiency (%)	Size (bp)
*AO*	TGGACGAGATGGGCAAGG	AAGCAGCGAAGCGGTGT	59	96	134
*DDC*	CGAGAACCAGGAGTCAG	CCAAACAGAAGGCAAAC	65	98	100
*IDO2*	GCTAACTGGAGGAAGAGG	AAGTCAGAAGATACCGTAA	58	95	294
*AOX1*	TGACGGAAGGATTGTTG	GTGAAGCTGTTACGGATG	57	94	112
*ALDH*	GAGGCTCTTTGCTGTCC	AGTTGTCCTCGGTCATAC	62	96	196
*DAO1*	GTCCCACAAAGTCAAGAT	ATTGACGAGACTGGCGA	64	98	176
*KYN*	ATCCGCCATTATTACAGC	ATCTTTGGGACCCTCGC	59	94	255
*KAT1*	AGTTCTTCGGTAGGATTG	GATAGTCGGTTACCACT	55	96	165
*CYP1A1*	CATCAACGAAGGCAAGA	GACAGCGATTACACTAGAC	54	99	255
*LAAO*	AGAAGCCAGTAACCGTATT	GACGAAAGGAAACGGAG	59	93	123
*β-actin*	GGCTACTCCTTCACCACCACA	TCTCCAAGGCAACGGGTCT	65	99	188

*AO*, amine oxidase; *DDC*, aromatic-L-amino-acid decarboxylase; *IDO2*, indoleamine 2,3-dioxygenase 2; *AOX1*, aldehyde oxidase 1; *ALDH*, aldehyde dehydrogenase; *DAO1*, amiloride-sensitive amine oxidase; *KYN*, kynureninase; *KAT1*, kynurenine–oxoglutarate transaminase 1; *CYP1A1*, cytochrome P450 family 1 subfamily A polypeptide 1; *LAAO*, L-amino-acid oxidase.

The β-actin is presented to be the internal control gene. QRT-PCR (Mastercycler ep realplex in Eppendorf, Germany) helped in detecting gene expression. The reaction environment of PCR and analysing approaches were identical, as mentioned above.

### Correlation analysis

To explore the correlation between downstream inflammatory genes and tryptophan metabolism, the crucial DEGs in the signal pathway of tryptophan metabolism were studied using canonical correlation analysis (CCA) with the inflammatory genes in internal tissue in the SBM40, SPC40, and FSBM40 groups, respectively. The key DEGs selected in the tryptophan metabolism signalling pathway include *AO, DDC, IDO2, AOX1, ALDH, DAO1, KYN, KAT1, CYP1A1*, and *LAAO*; the inflammatory genes include pro-inflammatory genes (*IL1β*, *TNFα*, and *IκBα*) and anti-inflammatory- related genes (*IL10* and *TGFβ1*) in the hindgut tissues.

### Statistics

With the help of free online omicshare tools, the CCA method was carried out to process the original data^1^. After homogeneity variance tests by SPSS 22.0 (SPSS Inc., Chicago, IL, USA), the results were studied with one-way ANOVA and are shown as the mean ± SD, with *P* < 0.05 indicating a statistically significant difference.

## Results

### Growth performance

As indicated in Table 4, the WGR and SGR of the experimental groups were lower than that of the FM, which served as the control group (*P* < 0.05). Among the experimental groups, there was little difference in WGR and SGR (*P* > 0.05). On the contrary, FCR increased significantly in each experimental group (*P* < 0.05). Among the experimental groups, there was little difference in FCR (*P* > 0.05). In addition, there was little difference in HSI and SR among the groups (*P* > 0.05).

**TABLE 4:** Effect of different soy meals at 40% substitution level for fish meal on the growth of grouper (*n* = 4).

Parameters	FM	SBM40	SPC40	FSBM40	*P*-value
IBW (g)	12.55 ± 0.00	12.55 ± 0.04	12.55 ± 0.02	12.55 ± 0.04	0.04
WGR (%)	485.14 ± 7.08^a^	426.50 ± 9.59^b^	431.73 ± 12.00^b^	427.22 ± 7.28^b^	0.03
SGR (%/day)	2.60 ± 0.02^a^	2.44 ± 0.03^b^	2.46 ± 0.03^b^	2.44 ± 0.02^b^	0.04
FCR	0.84 ± 0.01^a^	0.95 ± 0.02^b^	0.94 ± 0.03^b^	0.95 ± 0.02^b^	0.04
HSI (%)	2.43 ± 0.45	2.18 ± 0.25	2.36 ± 0.39	2.19 ± 0.19	0.02
SR (%)	99.17 ± 0.96	99.17 ± 0.96	99.58 ± 0.84	99.58 ± 0.84	0.03

IBW, initial body weight; FBD, final body weight; WGR, weight gain rate; SGR, specific growth rate; FCR, feed conversion ratio; HSI, hepatosomatic index; SR, survival rate.

^a,b^Values in the same row with different superscripts indicate significant differences (*P* < 0.05).

### Histological observations

**FIGURE 1:**
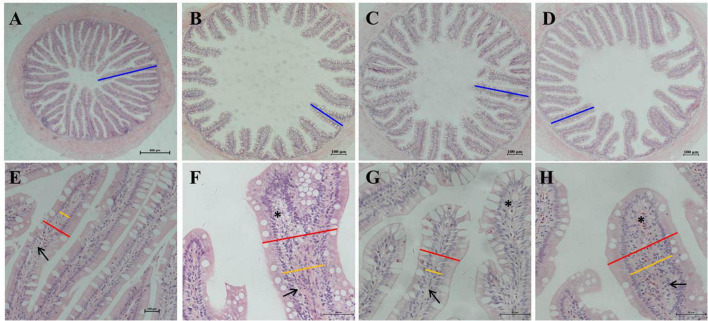
Hematoxylin-eosin staining in the hindgut of pearl gentian grouper. **(A–D)** Reduction and fusion of mucosal folds in SBM40, SPC40, and FSBM40 groups. **(E–H)** Increased width of lamina propria and inflammatory cell infiltration SBM40, SPC40, and FSBM40 groups. **(A,E)** Fish meal control group; **(B,F)** 40% SBM substitution for fish meal group; **(C,G)** 40% SPC substitution for fish meal group; **(D,H)** 40% FSBM substitution for fish meal group. Blue bar, the height of plica; red bar, the width of plica; yellow bar, the width of lamina propria; arrow, lamina propria; asterisk, inflammatory infiltration.

The H&E staining and semi-quantitative analysis showed that the experimental levels of SBM, SPC and FSBM all caused enteritis, and they presented significant distinctions compared to the control group (*P* < 0.05), among which the SBM40 and FSBM40 groups were more serious, and enteritis induced by SPC40 was significantly lighter than the SBM40 and FSBM40 groups (*P* < 0.05) (Figure 1 and Table 5).

**TABLE 5:** Semi-quantitative histological evaluation of gut sections (*n* = 10).

Parameters	FM	SBM40	SPC40	FSBM40	*P*-value
Mucosal folds	1.27 ± 0.12^a^	4.87 ± 0.15^b^	3.83 ± 0.35^c^	4.17 ± 0.55^d^	0.03
Lamina propria	1.47 ± 0.35^a^	4.77 ± 0.25^b^	3.97 ± 0.15^c^	4.50 ± 0.10^b^	0.01
Supranuclear vacuoles	1.77 ± 0.21^a^	4.80 ± 0.10^b^	3.77 ± 0.35^c^	4.13 ± 0.35^d^	0.03
Connective tissue	1.47 ± 0.25^a^	4.83 ± 0.21^b^	3.20 ± 0.36^c^	4.30 ± 0.46^b^	0.04

^a–d^Values in the same row with different superscripts indicate significant differences (*P* < 0.05).

### Physiological indexes determination

As exhibited in Table 6, IgM content, C3 content, and C4 content were all significantly reduced in hindgut tissues of all soy meal substitution groups (*P* < 0.05), while the T-SOD and LYS enzyme activities increased substantially in all soy meal substitution groups (*P* < 0.05).

**TABLE 6:** Effect of different soy meals at 40% substitution levels for fish meal on the enzyme activities of pearl gentian grouper (*n* = 3).

Parameters	FM	SBM40	SPC40	FSBM40	*P*-value
T-SOD (U/mg)	78.23 ± 9.95^a^	115.50 ± 12.07^b^	154.04 ± 13.35^c^	135.55 ± 14.25^c^	0.02
IgM (μg/mg)	94.33 ± 4.22^a^	64.70 ± 3.63^b^	37.77 ± 3.15^c^	50.84 ± 5.60^b^	0.03
C3 (μg/mg)	85.58 ± 5.31^a^	61.70 ± 8.21^b^	39.97 ± 7.49^c^	51.34 ± 7.73^b^	0.03
C4 (μg/mg)	128.83 ± 10.17^a^	92.88 ± 5.62^b^	60.32 ± 5.23^c^	76.83 ± 8.28^d^	0.01
LYS (U/g)	5.45 ± 0.47^a^	7.95 ± 0.64^b^	7.64 ± 0.64^b^	4.13 ± 0.86^c^	0.02

T-SOD, total superoxide dismutase; IgM, immunoglobulin M; C3, complement 3; C4, complement 4; LYS, lysozyme. ^a–d^Values in the same row with different superscripts indicate significant differences (*P* < 0.05).

### Immune-related gene expressions

As presented in Table 7, except for the gene expressions of p65, which significantly decreased (*P* < 0.05) and there was a little difference in *IκBα* (*P* > 0.05) in the SPC40 group, the pro-inflammatory gene expressions of *IL1β*, *IL8*, *TNFα*, *p65*, and *IκBα* significantly increased in all soy meal substitution groups (*P* < 0.05). The anti-inflammatory-related gene expressions of *IL5*, *IL10*, and *TGFβ1* were reduced considerably in all substitution groups (*P* < 0.05).

**TABLE 7:** Effect of different soy meals at 40% substitution levels for fish meal on the inflammatory-related gene expressions in hindgut of pearl gentian grouper (*n* = 3).

Gene	FM	SBM40	SPC40	FSBM40	*P*-value
*IL1β*	1.16 ± 0.16^a^	1.85 ± 0.12^b^	1.58 ± 0.09^c^	1.74 ± 0.23^c^	0.04
*IL8*	1.00 ± 0.08^a^	2.35 ± 0.17^b^	1.86 ± 0.16^c^	1.62 ± 0.04^c^	0.03
*TNFα*	1.01 ± 0.15^a^	1.52 ± 0.23^b^	1.33 ± 0.03^b^	4.09 ± 0.61^c^	0.01
*p65*	1.00 ± 0.10	1.59 ± 0.10^b^	0.42 ± 0.14^c^	1.63 ± 0.15^b^	0.02
*iκBα*	1.01 ± 0.14	2.20 ± 0.50^b^	0.95 ± 0.18^a^	2.49 ± 0.09^b^	0.02
*IL5*	1.00 ± 0.05^a^	0.71 ± 0.03^b^	0.55 ± 0.10^c^	0.40 ± 0.05^d^	0.03
*IL10*	1.03 ± 0.06^a^	0.36 ± 0.09^b^	0.56 ± 0.04^c^	0.56 ± 0.03^c^	0.04
*TGFβ1*	1.00 ± 0.08^a^	0.46 ± 0.16^b^	0.18 ± 0.05^c^	0.13 ± 0.01^c^	0.03

IL, interleukin; TNF, tumour necrosis factor; p65, NF-κB-p65; IκBa, I-kappa-B-alpha; TGF, transforming growth factor. ^a–d^Values in the same row with different superscripts indicate significant differences (*P* < 0.05).

### Sample diversity analysis of 16S sequencing

**FIGURE 2:**
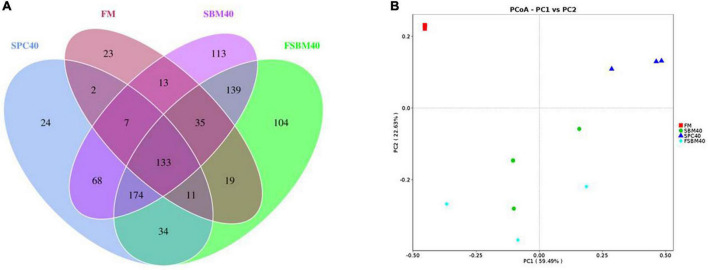
Venn plot **(A)** and PCoA **(B)** analysis of OTU by high-throughput sequencing after replacing fish meal with different soy meals in pearl gentian grouper (*n* = 3).

From the 12 samples, there were 983,965 original reads. The average amount of original reads of the FM, SBM40, SPC40, and FSBM40 groups were 79,339.67 ± 4,821.81, 86,036.67 ± 790.22, 81,344.00 ± 4,753.11 and 81,268.00 ± 4,575.00, respectively. After clustering into identical operational taxonomic units (OTUs) by sequences with similarity ≥97%, the researchers gained 899 OTUs. The average number of OTUs in the FM, SBM40, SPC40, and FSBM40 groups were 175.00 ± 32.79, 608.67 ± 56.84, 368.00 ± 42.58, and 717.33 ± 156.62, respectively. Except for a small significant difference between SBM40 and FSBM40 (*P* > 0.05), there were significant differences among the FM and SPC40 groups (*P* < 0.05). A Venn plot showed that 133 OTUs were among the groups (Figure 2A). The PCoA analysis revealed that different experimental groups could be clustered well, indicating that our samples were reasonably processed (Figure 2B).

### Comparison of gut flora composition and abundance

**FIGURE 3:**
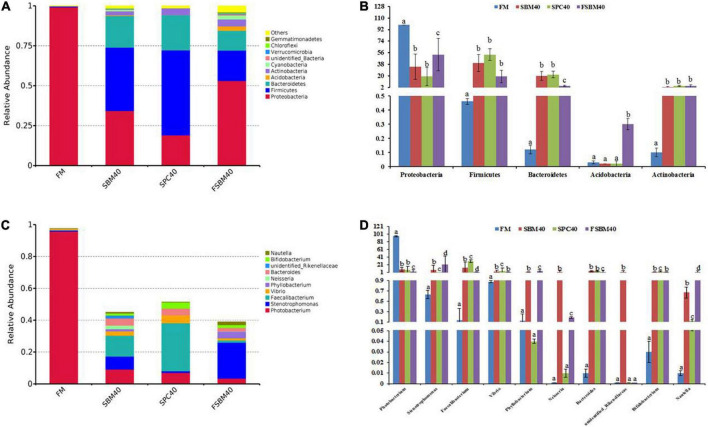
The gut flora composition and abundance of different soy meals substitution for fish meal in pearl gentian grouper [**(A,B)** phylum level and **(C,D)** genus level]. **(A)** Rarefaction curve; **(B)** rank abundance; **(C)** species accumulation boxplot. Different letters distributed on the column represented significant variances between the groups at *P* < 0.05 (*n* = 3).

The relatively abundant top six species at the phylum level are ranked as follows: (1) Proteobacteria, (2) Firmicutes, (3) Bacteroidetes, (4) Acidobacteria, (5) Actinobacteria, and (6) Cyanobacteria. The abundance of Proteobacteria significantly decreased in all soy meal substitution groups (*P* < 0.05), while the abundances of Firmicutes, Bacteroidetes, and Actinobacteria had a significant increase in all soy meal substitution groups (*P* < 0.05). There was little difference in acidobacteria abundance between the SBM40 group and the SPC40 groups, but it significantly increased in the FSBM40 group (Figures 3A,B).

The relatively abundant top six species at the genus level are ranked as follows: (1) *Photobacterium*, (2) *Stenotrophomonas*, (3) *Vibrio*, (4) *Phyllobacterium*, (5) *Neisseria*, and (6) *Bacteroides.* The abundance of *Photobacterium* experienced a significant decrease in all soy meal substitution groups (*P* < 0.05). The abundances of *Phyllobacterium* and *Neisseria* had little difference in the SPC40 group (*P* > 0.05), and the abundance of unidentified_*Rikenellaceae* had little difference between the SPC40 and FSBM40 groups (*P* > 0.05). The abundances of the left species significantly increased in all soy meal substitution groups (*P* < 0.05; Figures 3C,D).

### Statistics and KEGG enrichment of differential genes

**FIGURE 4:**
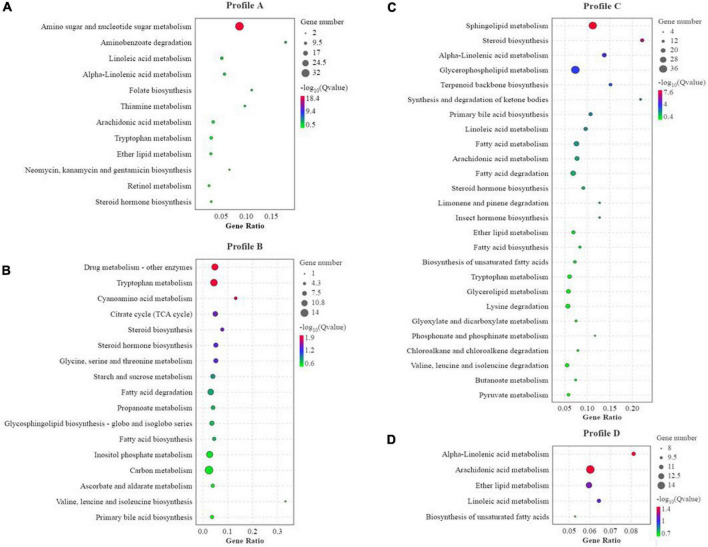
KEGG enrichment analysis of differential genes of different soy meals substitute for fish meal in hindgut of pearl gentian grouper (*n* = 4). **(A)** KEGG enrichment of co-contained DEGs in SBM40, SPC40, and FSBM40 group related to nutrition metabolism; **(B)** KEGG enrichment of unique DEGs in SBM40 group related to nutrition metabolism; **(C)** KEGG enrichment of unique DEGs in SPC40 group related to nutrition metabolism; **(D)** KEGG enrichment of unique DEGs in FSBM40 group related to nutrition metabolism (*n* = 4).

Compared to the FM group, there were 554 co-contained DEGs in the SBM40, SPC40, and FSBM40 groups, named profile A. The numbers of unique DEGs were 1,003, 2,254, and 1,656, named profile B, profile C, and profile D, respectively (Figure 4).

In profile A, the enrichment result showed that there were 238 pathways, of which 30 pathways indicated significant enrichment (*P* < 0.05). Among these pathways, there were 60 pathways relevant to nutrition metabolism, of which 12 pathways indicated significant enrichment (*P* < 0.05). Thus, within all the significant enrichment pathways, 40% (12/30) significant enrichment pathways were related to nutrition metabolism (Figure 4A). In profile B, the enrichment result showed that there were 297 pathways, of which 51 were significantly enriched (*P* < 0.05). Among these pathways, 81 pathways were relevant to nutrition metabolism, of which 17 pathways indicated significant enrichment (*P* < 0.05). In other words, within all the significant enrichment pathways, 33.33% (17/51) pathways were related to nutrition metabolism (Figure 4B). In profile C, the enrichment result showed 320 pathways, of which 35 were significantly enriched (*P* < 0.05). Among them, 98 pathways were relevant to nutrition metabolism, of which 26 indicated a significant enrichment (*P* < 0.05). Hence, within all the significant enrichment pathways, 74.3% (26/35) significant enrichment pathways were related to nutrition metabolism (Figure 4C). In profile D, the enrichment result showed that 305 pathways, of which 38 were significantly enriched (*P* < 0.05). Among them, 75 pathways were related to nutrition metabolism, of which six pathways indicated significant enrichment (*P* < 0.05). Thus, within all the significant enrichment pathways, 13.2% (5/38) were related to nutrition metabolism (Figure 4D). The tryptophan metabolism pathway was significantly affected in the experiments of all three soy meal substitutions for FM (*P* < 0.05).

### Validation of the tryptophan metabolism pathway by RT-qPCR

**FIGURE 5:**
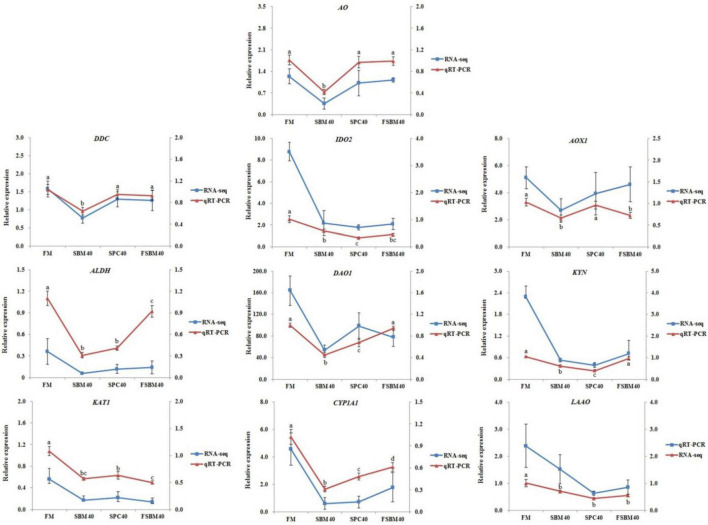
The contrast of RNA-seq and RT-qPCR results and the expressions of the key genes in tryptophan metabolism pathway in hindgut were chosen to confirm the precision of “3 + 2” transcriptome sequencing. The relative expression degree in RNA-seq analysis was counted by FPKM value. Different letters distributed on the broken line represented significant variances between the groups at *P <* 0.05 (*n* = 4).

**FIGURE 6:**
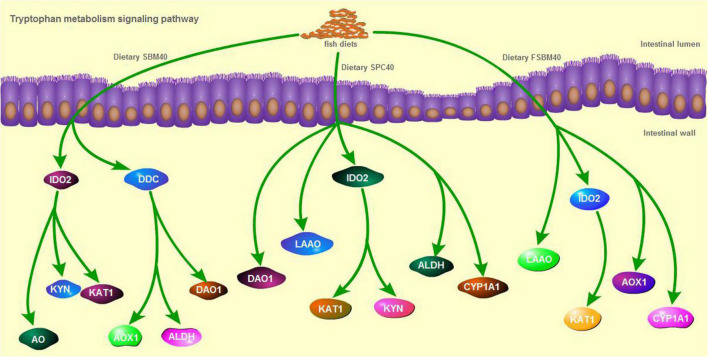
The key genes inhibited in tryptophan metabolism pathway of different soy meals induced enteritis in pearl gentian grouper.

In general, the results of RT-qPCR corresponded to the results of transcriptome sequencing data, which confirmed the accuracy of RNA-seq (Figure 5). Therefore, RNA-seq in this study can provide a relatively valuable reference for further analysis. The results of the RT-qPCR showed that the key genes selected from the tryptophan metabolism pathway in different soy meal substitution groups were generally significantly inhibited (*P* < 0.05). However, there were some differences in the types of key genes of the tryptophan metabolism pathway affected in different treatment groups (Figure 6).

### Correlation analysis

**FIGURE 7:**
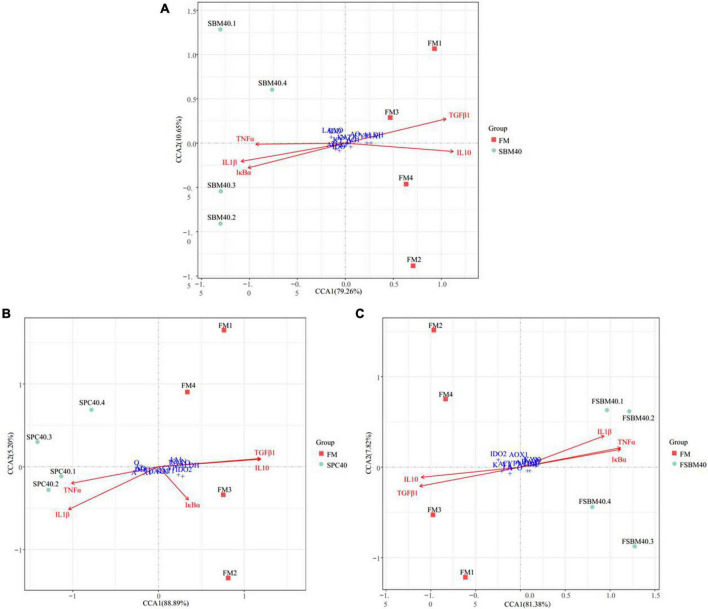
The canonical correlation analysis (CCA) between the key genes inhibited in tryptophan metabolism pathway and the inflammatory genes of different soy meals induced enteritis in groupers (*n* = 4). In CCA plot, the arrows represent explanatory variables and the points represent response variables. The lengths of the arrows represent the strength of the influence of the explanatory variable on the response variable; the longer lengths indicate greater influences. The angles between the arrows and coordinate axis represent the correlation between the explanatory variable and coordinate axis; smaller angles indicate stronger correlation. A sample point located in the same direction as the arrow indicates that the changes in explanatory and response variables are positively correlated, while in the opposite direction which indicates a negative correlation. The value in the coordinate axis label in the plot represents the interpretation proportion of the explanatory variables combination and the response variable variation. FM, fish meal control group; SBM40, 40% SBM substitution for FM; SPC40, 40% SPC substitution for FM; FSBM40, 40% FSBM substitution for FM.

Figure 7 shows the correlations between the significant genes in the inflammatory genes and tryptophan metabolism pathways using the Envfit test. The results show that with increases in SBM, the key genes inhibited in tryptophan metabolism pathways were generally significantly correlated with the enteritis-related inflammatory genes (*P* < 0.05, Supplementary Tables 1–3). Thus, it had a negative correlation with the anti-inflammatory gene and a positive correlation with the pro-inflammatory gene.

## Discussion

The present study exhibited that different soy meals (SBM, SPC, and FSBM) substitution for FM at the experimental level obviously lowered the growth performance, gut physiology and immune state, and gut flora composition. Studies on *Epinephelus coioides* (initial weight of 84 ± 2.5 g) found that 20% SBM substitution for FM (60% basal FM) presented optimal growth performance (10). Studies on the brown-marbled grouper (initial weight of 6.1 ± 0.7 g) showed that using 20–50% SBM to substitute for FM (50% basal FM) was unlikely to lower growth performance, but it significantly decreased at a 60% substitution level (12). SPC with substitution levels lower than 40% presented a significant reduction in the WGR, SGR, and FCR of turbot and Japanese flounder (28, 29). The optimal amount of FSBM in the hybrid tilapia diet substitution for FM is 34.2% (30), and FSBM in channel catfish diet can substitute 100% FM, but 25% is best (31). The present study found that WGR and SGR at a high level of substitution (40%) were significantly lower than in the FM control group. A previous study indicated that the soy protein replacing FM in fish growth physiology may be related to the ANFs it contains (3). SBM, SPC, and FSBM, as three soy protein sources with different processing forms, have different content and types of ANFs. The unpublished feed test results in the present research showed that alcohol-soluble ANFs in SPC were basically completely removed, such as soybean isoflavones and (7S and 11S) and conglycinin. Although FSBM was fermented, compared with the SBM diet, the content of soybean isoflavones and (7S and 11S) conglycinin and other ANFs showed little change. However, compared with SBM and FSBM, the pearl gentian grouper fed with SPC feed did not show obvious advantages. It is speculated that ANFs may not be the only main influencing factor, but they may also be the influence of other unknown antigens or non-alcohol-soluble ANFs (such as phytic acid). FSBM did not improve the growth performance of pearl gentian groper, which may be due to its high content of ANFs or palatability problems (32). Previous studies on *E. coicoiaes* found that adding more than 14% FSBM to the diet would significantly reduce WGR and SGR, and the most suitable amount of FSBM to replace white FM (52% of basal FM) was 10% (10).

The present research also indicated that for pearl gentian groupers, the experimental level of soy proteins also had a significant impact on their gut enzyme activity. The LYS plays an important role in non-specific immunity in fish and IgM for specific immunity. Both C3 and C4 are crucial for activating their complement systems (33). In the research, all the soy protein sources with a 40% substitution level reduced the contents of C3, C4, and IgM, in which the addition of soy proteins may lead to the impaired gut immune system of pearl gentian groupers. This is also reflected in immune-related genes, such as inflammatory-related genes. In addition, previous research confirmed that the occurrence of enteritis in fish always goes with down-regulation of anti-inflammatory genes and up-regulation of pro-inflammatory genes (34). The present experiment found similar results.

Our previous analysis of the gut immune system of SBMIE pearl gentian groupers found that among the signal pathways significantly activated in the gut, 55.17% were related to intestinal immunity, contagious disease, and signal transduction; in the signal pathways greatly inhibited in the gut, nutrient metabolism-related pathways accounted for 67.44% (35). The results showed that for pearl gentian groupers, the production of SBMIE is likely to be the result of the combined action of immune and nutritional imbalance. Combined with this study, we found different gut responses of SBMIE pearl gentian groupers that ate SPC and FSBM. After eating SPC, grouper mainly showed that the pathways relevant to intestinal nutrient metabolism were restrained in general (74.3%, 26/35), while after eating FSBM, the pathways related to immunity, contagious disease, and signal transduction were generally inhibited (60.5%, 23/38). Among them, the tryptophan metabolism pathway is significantly inhibited in the SBMIE pearl gentian groupers caused by the studied soy protein sources in the present study. According to existing research, tryptophan, as a communication medium between gut flora and the host, generates many tryptophan metabolites under the action of a series of endogenous enzymes or biological metabolisms, and its metabolites regulate various physiological processes of the host to varying degrees. Tryptophan deficiency directly leads to the reduction of metabolites, which is closely related to gut imbalance, and even induced IBD (36).

Tryptophan is the only amino acid in animals that combines with serum protein through covalent bonds. It is widely involved in the synthesis of proteins and nucleic acids, and it can enter the liver through the hepatic portal vein and regulate protein deposition and metabolism. Insufficient tryptophan content will reduce the absorption and utilisation rate of protein and immune function of livestock and poultry and increase the susceptibility of livestock and poultry to disease (37, 38). There are three main pathways of tryptophan catabolism: the kynurenine pathway, 5-hydroxytryptamine (5-HT) pathway and the gut flora pathway. The kynurenine pathway is the most important metabolic pathway through which about 95% of tryptophan is metabolised (39, 40). This pathway occurs in three parts of the body, which, in order of degree, are the liver, brain, and small intestine. The rate-limiting enzymes are TDO and –2,3–dioxygenase IDO, respectively (41). Tryptophan is catalysed by a series of enzymes, such as TDO/IDO to produce a variety of metabolites, such as N-formyl kynurenine, kynurenine, 3-hydroxykynurenine, kynurenic acid, and picolinic acid, which are finally decomposed into CO_2_ and ATP (42). After being absorbed by the body, these metabolites can regulate intestinal barrier immunity in a variety of ways. Second, 1–2% of tryptophan intake is converted through the 5-HT pathway, mainly in the gastrointestinal tract, of which 90% occurs in enterochromaffin cells and 10% in gut neurons, and the rate-limiting enzymes are tryptophan hydroxylase (43). 5-HT not only regulates the immune activity of the body, but it also plays a key role as a neurotransmitter in the brain-gut axis, which is a bidirectional system that connects the gastrointestinal tract with the central nervous system (44). In addition, 4–6% of tryptophan can be directly metabolised by intestinal flora into ligands of aromatic hydrocarbon receptor (AhR), including indole and indole derivatives (45, 46). In terrestrial animal studies, it is pointed out that AhR can bind to its ligand through TLR-NF-κB signalling pathway, and then it regulates the expression of downstream target genes. For example, studies in mammals have found that a tryptophan-rich diet can activate the expression of the AhR gene and subsequently increase the expression of the IL22 gene in the colon (47). AhR is a negative regulator of IL17-mediated signal transduction and plays an important role in resisting pathogens of immune diseases (48). IL10 is an effective anti-inflammatory cytokine that can inhibit the production of pro-inflammatory mediators. IL10 signals through IL10 receptors, which act on various cell types, such as intestinal epithelial cells, where IL10 receptors are associated with the development and maintenance of barrier function (49). In fish SBMIE, NF-κB signalling pathway presents to be quite conservative, and similar results were obtained in our previous analysis of pearl gentian groupers enteritis induced by SBM and FSBM (35). In this experiment, through CCA analysis, we found that there was a significant correlation between the key genes of the tryptophan metabolism pathway and enteritis induced by three soy protein sources of pearl gentian grouper, which also suggested that abnormal tryptophan metabolism in fish soy enteritis deserves special attention.

Some studies have pointed out that the gut flora variation will lead to a change in tryptophan metabolism and then affect the intestinal physiological function (50). Similarly, tryptophan deficiency in feed can also change the composition of gut flora and damage intestinal immune function (51). Previous research has pointed out that the tryptophan content in SBM is generally equivalent to that in FM (∼0.65%), while the tryptophan content in SPC and FSBM is higher than that in FM (52). However, the three kinds of soy protein feeds in this research have led to the inhibition of tryptophan metabolism, which is likely to be closely related to gut flora variation. The three soy protein sources at this experimental level significantly influenced the intestinal flora of pearl gentian groupers. In mammalian studies, it was found that the abundance changes in Bacteroides, Bacteroides, Bifidobacterium, Fusobacterium, Lactobacillus, and Faecalibacterium (53–55) were significantly correlated with the degree of IBD. Similarly, the abundance changes in some species at the level of gut flora were also found in the SBMIE pearl gentian grouper, but the change trend was opposite. Previous research pointed out that under pathological state, the OTU abundance variations of mammalian gut flora at the level of the phylum or genus run contrary to those of fish sometimes (56–58). At present, the analysis of intestinal flora in this experiment is only at the genus level, and it should be further studied at the species level in the future.

## Conclusion

This study found that the tryptophan metabolic pathway has an important impact on the enteritis of pearl gentian groupers induced by soy protein, during which gut flora may play a crucial role. However, a more in-depth analysis should be further improved in future work. Technically, targeted metabonomics can be used to analyse the changes of tryptophan metabolites, such as indoles and indole derivatives produced by gut flora metabolism; using metagenomics to analyse the species of tryptophan metabolising flora in the gut at the species level, as a potential probiotic additive for repairing fish soy-induced enteritis. The research offers a theoretical reference for the development of new feed additives to prevent soy-induced enteritis in fish and has important economic and ecological benefits.

## Data availability statement

The datasets presented in this study can be found in online repositories. The names of the repository/repositories and accession number(s) can be found in the article/Supplementary material.

## Ethics statement

All experimental methods were approved by the Ethics Review Board of Guangdong Ocean University. All of the procedures were performed in accordance with the relevant guidelines and regulations.

## Author contributions

WZ and AP were engaged in the whole experiment and formulated the draft of this manuscript. BT designed the experiment and composed and amended the draft critically. YX and YL took part in the tests. RX amended the first draft. HZ and QY completed the data analysis. JD and SC approved the final version. All authors contributed to the article and approved the submitted version.

## Footnotes

1
https://www.omicshare.com/tools
