# Supplementary material for: Tryptophan metabolism and gut flora profile in different soybean protein induced enteritis of pearl gentian groupers
Source: Front Nutr. 2022 Dec 19;9:1014502. doi: 10.3389/fnut.2022.1014502 (PMC9807032; doi:10.3389/fnut.2022.1014502)
Supplement: Supplementary file 1 [file Data_Sheet_1.docx]

**Supplementary Table 1** The CCA analysis between key genes with significant variarions in tryptophan metabolism pathway and inflammatory gene expressions in hindgut of pearl gentian grouper by SBM induced enteritis of (*n* = 4)

| Gene | CCA1 | CCA2 | r2 | *P* |
| --- | --- | --- | --- | --- |
| *IL1β* | -0.9824 | -0.1869 | 0.9417 | 0.0439 |
| *TNFα* | -0.9999 | -0.0126 | 0.6663 | 0.0225 |
| *iκBα* | -0.9641 | -0.2656 | 0.8507 | 0.0433 |
| *IL10* | 0.9965 | -0.0837 | 0.9815 | 0.025 |
| *TGFβ1* | 0.9676 | 0.2524 | 0.9045 | 0.0319 |

**Supplementary Table 2** The CCA analysis between key genes with significant variarions in tryptophan metabolism pathway and inflammatory gene expressions in hindgut of pearl gentian grouper by soybean protein concentrate induced enteritis of (*n* = 4)

| Gene | CCA1 | CCA2 | r2 | *P* |
| --- | --- | --- | --- | --- |
| *IL1β* | 0.9512 | 0.3087 | 0.9293 | 0.0181 |
| *TNFα* | 0.9994 | 0.0253 | 0.7585 | 0.0481 |
| *iκBα* | -0.5165 | 0.8563 | 0.1849 | 0.7458 |
| *IL10* | -0.9998 | 0.0198 | 0.9796 | 0.0486 |
| *TGFβ1* | -0.9986 | 0.0535 | 0.9731 | 0.0386 |

**Supplementary Table 3** The CCA analysis between key genes with significant variarions in tryptophan metabolism pathway and inflammatory gene expressions in hindgut of pearl gentian grouper by fermented soybean meal induced enteritis of (*n* = 4)

| Gene | CCA1 | CCA2 | r2 | *P* |
| --- | --- | --- | --- | --- |
| *IL1β* | 0.9378 | 0.3472 | 0.7509 | 0.0408 |
| *TNFα* | 0.9849 | 0.1731 | 0.9769 | 0.0181 |
| *iκBα* | 0.9821 | 0.1884 | 0.9829 | 0.0333 |
| *IL10* | -0.9949 | -0.1011 | 0.938 | 0.0501 |
| *TGFβ1* | -0.9829 | -0.1843 | 0.9918 | 0.0222 |
