# Supplementary material for: Tryptophan metabolism and gut flora profile in different soybean protein induced enteritis of pearl gentian groupers
Source: Front Nutr. 2022 Dec 19;9:1014502. doi: 10.3389/fnut.2022.1014502 (PMC9807032; doi:10.3389/fnut.2022.1014502)
Supplement: Supplementary file 2 [file Table_1.DOCX]

**Supplementary File 1**

**The detailed procedures of omics sequencing**

**16S analysis of intestinal microflora**

*Genome DNA extraction and amplification*

Total RNA of pearl gentian grouper DI microflora was extracted by using E.Z.N.A.^TM^ Kit (Omega Bio-Tek, Norcross, GA, U.S.) according to the instructions. The concentration and purity testing of extracted genomic DNA were tested by using 1% agarose gel. Then the DNA was diluted to 1 ng/μL using sterile water according to its concentration. The above DNA templates were amplified by primers 338F: ACTCCTACGGGAGGCAGCA and 806R: GGACTACHVGGGTATCTAAT of V3-V4 region of bacterial16SrDNA gene. All PCR reactions were carried out in 30 μL reaction system, using 15 μL Phusion® High-Fidelity PCR Master Mix（New England Biolabs), 0.2 μm forward and reverse primers, and about 10 ng template DNA. The PCR reaction conditions were as follows: pre-denaturation at 98 ^o^C for 1 min, denaturation at 98 ^o^C for 10 s, annealing at 50 ^o^C for 30 s, extension at 72 ^o^C for 30 s, 30 cycles, and extension was at 72 ^o^C for 5 min.

*PCR product purification*

The PCR product was mixed with an equal volume of 1 × loading buffer (containing SYB green) and detected by 2% agarose gel electrophoresis. The sample was mixed equally according to the concentration of PCR product. Then, purification was performed using the Gene JET^TM^ Gel Ectraction Kit (Thermo Scientific).

*Library construction and sequencing*

According to the operation instructions, the library was constructed with Ion Plus Fragment Library Kit 48 rxns Kit (Thermo Scientific). After quantification and detection by Qubit2.0 Fluorometer Kit (Thermo Scientific), the library was sequenced using IonS5^TM^XL platform and 400 bp/600 bp single-end were generated. The original reads are stored in the NCBI sequential read Archive (SRA) database and the accession number is PRJNA666309.

*Data processing and analysis*

Exporting IonS5TMXL offline data to fastq file. The data of each sample is distinguished according to the barcode sequence. In order to obtain high-quality and effective data (clean reads), Cutadapt software (V1.9.1, http://cutadapt.readthedocs.io/en/stable/) is used to filter the raw data. Subsequently, the preliminary filtered reads were compared to the Silva database using UCHIME algorithm (UCHIME Algorithm, http://www.drive5.com/usearch/manual/uchime_algo.html) to detect the chimeric sequence, and then the chimeric filtering is carried out to obtain the effective data that can be used for subsequent analysis, i.e. clean reads. After that, Uparse software (http://drive5.com/uparse/) was used to cluster all clean reads of all samples, and the sequences with similarity ≥ 97% were clustered into the same Operational Taxonomic Units (OTUs). Finally, Mothur algorithm (version 1.39.1, http://www.mothur.org/) and Silva database (https://www.arb-silva.de/) were used for species annotation analysis of OTUs sequences.

**Transcriptome analysis**

*RNA extraction and detection*

Total RNA of pearl gentian grouper DI tissues were extracted using Trizol Kit (Invitrogen, USA) following manufacturer's instructions. First, genome DNA was removed using DNaseI (Invitrogen, USA). Then, 1% agarose gel electrophoresis was used to analyze RNA degradation degree and whether there was contamination. Subsequently, Nanodrop 2000 (Thermo Fisher Scientific, USA) was used to detect RNA purity (OD260/280 ratio). Finally, Qubit was used to accurately quantify RNA concentration, and Agilent 2000 (Agilent Technologies, USA) was used to accurately test RNA integrity.

*Library construction*

(1) Third-generation library construction and sequencing

The mRNA was enriched by the magnetic beads which contain Oligo (dT) (Tiangen, China). The mRNA was reversed transcription the mRNA into cDNA using SMARTer PCR cDNA Synthesis Kit. PCR was used to amplify and enrich the synthesized cDNA, and the optimal PCR conditions were determined by cycle optimization. Partial cDNA was screened by BluePippin and enriched with more than 4 kb fragments, and large scale PCR was carried out to obtain enough total amount of cDNA. the full-length cDNA was used for damage repair, end repair, and connection of SMRT dumbbell shaped connector, and the equimolar library of non-screened fragments and fragments larger than 4kb was constructed; exonuclease digestion was used to remove the sequence of unconnected junctions at both ends of cDNA; finally, a complete SMRT bell library was constructed by binding primers and DNA polymerase. After passing the library inspection, the library was sequenced by PacBio Sequel platform according to the effective concentration of the library and data output requirements. The raw PacBio SMRT sequencing raw reads and Illumina sequencing raw reads are deposited in NCBI Sequence Read Archive (SRA) and the accession numbers are PRJNA664623 and PRJNA664416, respectively.

(2) Second-generation library construction and sequencing

After total RNA was extracted, the mRNA was enriched by Oligo(dT) beads, while the mRNA was enriched by removing rRNA by Ribo-Zero^TM^ Magnetic Kit (Epicentre). Then, the enriched mRNA was fragmented into short fragments using fragmentation buffer and reverse transcripted into cDNA with random primers. Second-strand cDNA was synthesized by DNA polymerase I, RNase H, dNTP and buffer. Then the cDNA fragments were purified with QiaQuick PCR extraction kit, end repaired, poly(A) added, and ligated to Illumina sequencing adapters. The ligation products were size selected by agarose gel electrophoresis, PCR amplified, and sequenced using Illumina HiSeq^TM^ 4000 by Gene Denovo Co., Ltd. (Guangzhou, China).

*PacBio SMART data processing*

After the sequence completing, the offline raw data are de spliced and read with low quality. The output is filtered and processed by the software SMRTlink V5.1. The parameters are: - minlength = 200, - minreadscore = 0.65, and then the final data is the valid data. In order to obtain the full-length transcripts, first, the subreads sequence was self-corrected to form CCS (parameter: - minpasses = 2, minpredicted accuracy = 0.8), and high quality transcript consistent sequence was obtained. The non-chimeric sequence with 5' primer, 3' primer and PolyA tail is called full-length non-chimeric sequence (FLNC). IEC algorithm was used to cluster the FLNC sequences of the same transcript to obtain CCS, and then non full length sequences were used to correct the CCS. Then, the fused consensus sequences (CS) were obtained for subsequent analysis. After that, the Illumina RNA sequencing data were used to correct the polished consensus sequence by LoRDEC software (parameters: -k21, -s3) to further improve the accuracy of sequencing. Finally, CD-HIT-v4.6.7 (-c0.95 -T6 -G0 -aL0.00 -aS0.99) software was used to cluster and compare protein or nucleic acid sequences by sequence alignment, and remove redundant and similar sequences.

*Analysis of differentially expressed genes (DEGs)*

Clean reads obtained from Illumina sequencing were mapped to SMART sequence library by hisat2v2.05 software. RSEM software was used to calculate the gene expression level of each sample. The read count of each gene was obtained from the comparison results and converted into FPKM value. The DEGs were identified by DESeq R software package. Among them, the genes with |log2FC| > 1 and *P* < 0.05 were identified as DEGs. On the basis of identification of differential genes, the DEGs analysis was carried out in this study. The DEGs genes with significant differences (*P* < 0.05) were annotated with KEGG, and the signal pathways with significant differences related to nutrition metabolism were further analyzed (*P* < 0.05).

*Validation of real-time quantitative PCR and Western blotting*

In order to validate the accuracy of RNA-seq data, the RNA samples stored at -80 ^o^C for transcriptome sequencing were selected for RT-qPCR analysis. In this research, 18 genes related to inflammation were selected, including *AO*, *DDC*, *IDO2*, *AOX1*, *ALDH*, *DAO1*, *KYN*, *KAT1*, *CYP1A1* and *LAAO*. The primer design, synthesis and sequence source of all the genes are the same as those mentioned above, and showed in Table 3. The internal control gene is *β-actin*. The expression levels of these genes were detected by RT-qPCR. The PCR reaction conditions were 95 ^o^C for 2 min, 1 cycle; followed by 40 cycles of 95 ^o^C for 15 s, 60 ^o^C annealing for 10 s, and 72 ^o^C for 20 s. All reactions were done in triplicates. Melting curve analysis was performed to determine the target specificity. The RT-qPCR data were calculated using the 2^-ΔΔCT^ method.
